# The effects of high-frequency rTMS over the left DLPFC on cognitive control in young healthy participants

**DOI:** 10.1371/journal.pone.0179430

**Published:** 2017-06-14

**Authors:** Yanmin Li, Lin Wang, Meng Jia, Jihong Guo, Huijun Wang, Mingwei Wang

**Affiliations:** 1 Department of Neurology, The First Hospital of Hebei Medical University, Shijiazhuang, Hebei Province, China; 2 Brain Aging and Cognitive Neuroscience Laboratory of Hebei Province, Shijiazhuang, Hebei Province, China; 3 Department of Neural Electrophysiology, The First Hospital of Hebei Medical University, Shijiazhuang, Hebei Province, China; University Medical Center Goettingen, GERMANY

## Abstract

A large body of evidence suggests that repetitive transcranial magnetic stimulation (rTMS) is clinically effective in treating neuropsychiatric disorders and multiple sessions are commonly used. However, it is unknown whether multiple sessions of rTMS improve cognitive control, which is a function of the neural circuitry of the left dorsolateral prefrontal cortex (DLPFC)-cingulate cortex in healthy individuals. In addition, it is still unclear which stages of neural processing are altered by rTMS. In this study, we investigated the effects of high-frequency rTMS on cognitive control and explored the time course changes of cognitive processing after rTMS using event-related potentials (ERPs). For seven consecutive days, 25 young healthy participants underwent one 10-Hz rTMS session per day in which stimulation was applied over the left DLPFC, and a homogeneous participant group of 25 individuals received a sham rTMS treatment. A Stroop task was performed, and an electroencephalogram (EEG) was recorded. The results revealed that multiple sessions of rTMS can decrease reaction time (RTs) under both congruent and incongruent conditions and also increased the amplitudes of both N2 and N450 compared with sham rTMS. The negative correlations between the mean amplitudes of both N2 and N450 and the RTs were found, however, the latter correlation were restricted to incongruent trials and the correlation was enhanced significantly by rTMS. This observation supports the view that high-frequency rTMS over the left DLPFC can not only recruit more neural resources from the prefrontal cortex by inducing an electrophysiologically excitatory effect but also enhance efficiency of resources to deploy for conflict resolution during multiple stages of cognitive control processing in healthy young people.

## Introduction

Repetitive transcranial magnetic stimulation (rTMS) has become a powerful and non-invasive tool for improving the cognitive functions of the brain. The key feature of rTMS is its magnetic field, which induces an electric field that is sufficient to stimulate neurons within a short period of time [1]. rTMS can elicit an excitatory or inhibitory effect on neural circuitry [2]not only at the stimulation site beneath the coil but also in areas of the brain that are not directly under the coil [3, 4]. rTMS has been utilized to study a variety of cerebral functions in patients with various types of brain disorders, such as depression and dementia [5, 6] and multiple sessions of rTMS are frequently used. rTMS is not only clinically effective in treating neuropsychiatric disorders but also enable patients with these diseases to achieve better performance in various cognitive tasks, such as word recall [6], verbal memory [7], association memory [8], selective attention [9], language performance [10] and other executive functions [11]. Many studies have demonstrated that these improvements in cognition were maintained for 8 weeks to 3 months after several days of rTMS treatment [12].

In recent years, the effects of rTMS on cognition in healthy individuals have garnered increasing interest. For example, healthy young females who received one session of high-frequency (10 Hz) rTMS over the left dorsolateral prefrontal cortex (DLPFC) performed better in a Stroop task than those who received sham stimulation [13]. A similar improvement was observed by Hwang [14] who found that healthy young males showed fewer commission errors in a Conners' continuous performance test after rTMS. However, Rounis E [15] found that there was no improvement in attentional reorienting after one session of HF-rTMS applied over the left DLPFC in healthy volunteers. Thus it can be seen that previous studies included exclusively single-session stimulations and the results of rTMS on cognition are inconsistent. Generally, it was believed that longer stimulation trains would induce more lasting effects [16]. Little is known as to whether multiple sessions of HF-rTMS (commonly used in the treatment of neuropsychiatric disorders) would lead to similar improvements in heathy participants. Researchers applied multiple sessions of HF-rTMS in older adults undergoing normal aging and assessed cognition in a previous study [17]. However, there are no studies that focus on the effects of multiple sessions of HF-rTMS on cognition in young healthy people. Moreover, only males or females were included, a within-subject crossover design was used in many previous studies and the long-term effects of rTMS were not taken into account. Therefore, in the current study, we attempted to examine the effects of HF-rTMS over the left DLPFC and its potential mechanisms on cognitive control in young healthy participants. To avoid the limitations noted above, our study met the following requirements: 1) multiple sessions of HF-rTMS were used; 2) both young healthy males and females were involved; and 3) a randomized, sham rTMS controlled, between-groups design was adopted and possible carryover effects were excluded.

The biological mechanisms underlying the effects of rTMS on neuropsychiatric disorders and cognition are very complicated and remain largely unclear. Previous studies have suggested that blood flow [18] and metabolic changes [19] at the stimulation site, brain-derived neurotrophic factor upregulation [20], improvements in synaptic plasticity [21] and changes in the activity of the neural circuitry of the DLPFC-cingulate cortex, including both the DLPFC and the anterior cingulate cortex (ACC) [22–24], are involved. Event-related potentials (ERPs) are useful for the examination of cognitive processing variations because this method provides millisecond-level temporal resolution of neural activity. ERPs have been applied in many rTMS-related studies and have aided the investigation of rTMS effects on the time course of cognitive processing. For example, the latencies of target discrimination-related P3 were significantly shortened by high-frequency rTMS (20 Hz) over the left prefrontal cortex in a visual oddball paradigm [25] and a dual-task paradigm [26]. However, it is unclear at which stages during the time course of neural processing that cognitive control is affected, and how these stages are altered by rTMS.

Cognitive control, a key aspect of executive functioning, is closely related to mental health and consists of a series of multiple cognitive stages of perceptual processing, attention capture, conflict monitoring and conflict interference resolution among others[27]. The neural circuitry of the left DLPFC-cingulate cortex is the functional basis of cognitive control [28] according to the cognitive control theory which posits that the ACC detects a conflict in performance or the environment and, subsequently, signals the DLPFC to recruit control resources [29]. The Stroop task, stop-signal task, Eriksen flanker task and Go/NoGo task are among the tasks that are commonly used to evaluate cognitive control. From an electrophysiological standpoint, N2 and N450, which belong to the ACC-mediated conflict-monitoring family of ERPs [30], are two important components for the evaluation of cognitive control.

The N2 component has an anterior scalp distribution and peaks between 200 ms and 350 ms after stimulus onset. This component is generally interpreted as an index of conflict monitoring [31] or as a sign of a general control process that occurs in many situations, such as tasks that involve competing responses [32, 33]. According to the conflict monitoring theory, N2 amplitudes are more negative for incongruent trials relative to congruent trials [33]. According to other reports in the literature, however, rather than the amplitude of N2 reflecting the degree of conflict of a stimulus, N2 may represent the strategic deployment of cognitive control [34, 35]. The view that N2 represents the strategic deployment of cognitive control is supported by the findings obtained by Bartholow [36], who found that the N2 amplitude is sensitive to the probability of congruent and incongruent trials (and is largest for highly probable stimuli) but not with incongruent stimuli. Previous studies have suggested that control-related N2 is one of the indices of ACC activation [37]. N450 is a well-characterized negativity that peaks within the range of 350–550 ms during the color naming of incongruent color words compared with congruent color-word stimuli in the Stroop color-word task [38, 39]. Dipole source analysis suggests that this negative component is also generated in the ACC [38] and is most likely related to the processing of conflict monitoring as well as sensitive to incongruency [30].

rTMS is a promising tool for the enhancement of cognitive control. The results of previous studies have suggested that a single session of rTMS decreases reaction time (RT) in the Stroop task of healthy participants [13, 14]. ERP data have indicated that the amplitudes of the error-related negativity (ERN) and subsequent error-positivity (Pe) components were also altered in a typical Eriksen flanker task after rTMS [40]. However, other studies did not find any significant changes in either behavioral measures or ERP data associated with the Stroop task after rTMS [41, 42]. These discrepant results for the effects of rTMS on cognitive control may be due to the different rTMS parameters that were used in these studies, such as the stimulation type, location, frequency, and intensity or treatment duration. It is generally believed that low-frequency (<1 Hz) rTMS is likely to inhibit neuronal firing in a localized area, whereas high-frequency (>1 Hz) rTMS leads to neuronal depolarization, causing an excitatory effect under the stimulating coil [2], and is more likely to result in a significant neural enhancement and/or cognitive improvement. According to the previous literature [43], healthy participants tend to exhibit smaller improvements than patients after rTMS treatment. As mentioned above, longer stimulation trains would induce more lasting effects [16]. In other words, more pulses, higher intensity and longer treatment durations mean better effects to some extent with the control of safety and adverse effects of rTMS. Therefore, multiple sessions of HF-rTMS were used in our study.

In summary, the present study aimed to investigate the effects of multiple sessions of high-frequency rTMS over the left DLPFC on cognitive control and changes in the time course of the dynamic processing of cognitive control in healthy participants using ERP measurements. High-frequency rTMS (10 Hz) was applied one session a day for seven consecutive days, and a classical Stroop color-word task was utilized before and after the rTMS treatment. Based on the excitatory effect of high-frequency rTMS, we expected that performance on the Stroop task would be improved and that the amplitudes of N2 and N450 would be larger in the rTMS group compared with the sham rTMS group.

## Methods

### Participants

Fifty healthy postgraduate students (24 males and 26 females) from the Hebei Medical University in China were involved in the present study from March to November 2016. The age range of the sample was 24–30 years (mean 26.8±1.43 years). Participants who met the following criteria were enrolled in the study: 1) scores on the two mood scales (24-item HAMD [44] and HAMA [45]) that were each less than seven and scores on the Mini Mental State Examination (MMSE) [46]and Montreal Cognitive Assessment (MoCA) [47], which are widely used to screen for mild cognitive impairment, that were each greater than 27; 2) no previous exposure to rTMS; 3) no contraindications to rTMS exposure (e.g., epilepsy); 4) no metal implants in any part of the body, e.g., cardiac pacemaker, metal implants in the head or neck region, fixation elements and artificial joints; 5) no tattoos on any part of the body; 6) no history of neurologic or psychiatric disease or other health problems; 7) right-handedness (based on self-report); 8) normal or corrected-to-normal vision and normal color vision as assessed by basic vision tests; 9) no major self-reported life events during the study period; and 10) no alcohol use throughout the procedure.

The participants were randomly assigned to the rTMS group (25 participants with a mean age of 26.6±1.15 years) or the sham rTMS group (25 participants with a mean age of 26.9±1.68 years) after ensuring that age and gender were matched across the two groups. Each group had 12 males and 13 females. The composition of the groups (rTMS or sham rTMS) was not revealed until the experiment was completed. We managed the data of participants by serial number, name, and age to prevent confusion.

The study was performed in accordance with ethical guidelines and received ethical approval from the Medical Ethics Committee of the First Hospital of Hebei Medical University. All participants provided written informed consent and were awarded with ¥150 following completion of the experiment.

### General procedures

This study reports the results obtained from a larger study that addressed the effects of rTMS on cognition. Initial data were obtained under three experimental paradigms: the classical Stroop color-word paradigm, Go/NoGo paradigm, and visual oddball paradigm. Only data from the first paradigm are presented in this study.

**Fig 1:**
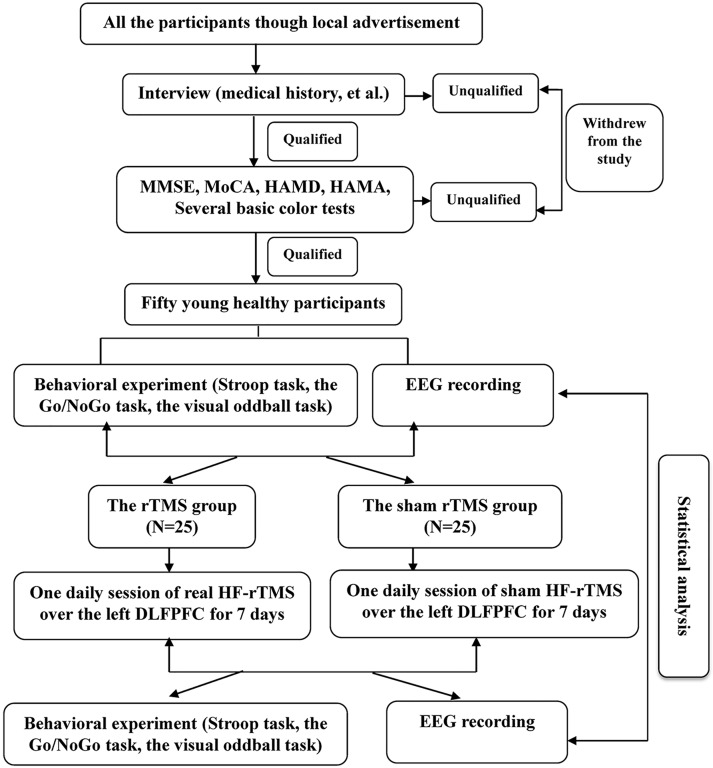
The Experimental Design Flow Chart of the study.

First, the participants were required to complete a set of questionnaires (described above) and several basic color tests. Following the successful completion of these qualifying procedures, the participants were fitted with 64 -scalp electrodes and seated in a comfortable chair in a dimly lit and electrically isolated room. The classical Stroop color-word task was administered, and the behavioral and electroencephalogram (EEG) data were collected prior to rTMS or sham rTMS administration. All of the initial data were collected 24 hours prior to the first administration of rTMS or sham rTMS. The Experimental Design Flow Chart is presented in Fig 1.

The participants received one daily session of rTMS or sham rTMS for seven consecutive days. After one week, the participants were immediately re-evaluated using the Stroop task and EEG recordings.

### rTMS procedure

rTMS was applied according to safety guidelines based on available safety studies of rTMS[48, 49]. Both real and sham rTMS were applied over the left DLPFC with a figure-eight-shaped coil, which induces a maximum electrical field that peaks beneath the intersection of the two windings [50]. All stimulations were performed with a MAGSTIM -high-speed stimulator (Magstim Company Limited, Wales, UK). The left DLPFC stimulation site was defined as being 5 cm anterior from the area of the optimal site for the primary motor cortex of the left hemisphere (the method of Pascual-Leone). This method has been reported to be accurate in targeting the DLPFC area [51]. The motor threshold (MT) was determined individually before the real and sham stimulations. The stimulation intensity was 110% of the motor threshold of the right abductor pollicis brevis muscle, and the stimulation frequency was 10 Hz. The other stimulation parameters were as follows: 45 trains of 3 s in duration were divided into 3 blocks, and the intertrain interval was 10s in each block. The interblock interval was 20 s, resulting in 1350 pulses per session for approximately 10.5 min per day. The real and sham rTMSs procedures were performed at the same location on the skull; however, for the sham stimulation, the figure-eight-shaped coil was held at a 90° angle, resting on the scalp with only one edge. Each individual received stimulation at the same time of day for all sessions (sessions were performed from either 08:00–09:00 or 14:00–15:00).

### Stimuli

Stimuli were shown on a 17-inch computer screen with a black background. Chinese color words were presented with Song Ti 96, which was viewed from a distance of approximately 70 cm. These characters appeared in the center of the screen and subtended a visual angle of approximately 2.39° horizontally and 2.99° vertically.

### Stroop color-word task

Each participant completed a computerized classical Stroop color-word task in Chinese characters. The stimuli consisted of congruent and incongruent stimuli, and the probability of either stimulus being presented was 50%. The congruent stimuli consisted of four color words in Chinese characters (red, yellow, green, blue) written in the same color in which the stimulus was presented (e.g., the word “red” in a Chinese character was written in red ink), with a probability of 12.5% of each word appearing at any time. The incongruent stimuli consisted of the same four words, but the display color did not match the word (e.g., the word “green” was written in red ink). Incongruent stimuli appeared at equal frequencies.

At the beginning of each trial, a fixation cross was presented for 500 ms, followed by the color word, which remained on the screen for 3000 ms or until the participant provided a response. The participants were asked to respond as fast and accurately as possible and to identify the printed color while ignoring the semantic meaning of each word. Four keys on a standard keyboard were used to enter their responses, i.e., using the keys “Z” (red color, left middle finger), “X” (yellow color, left index finger), “N” (blue color, right middle finger), and “M” (green color, right index finger). The response keys were counterbalanced between participants. The trials concluded with a variable inter-trial interval that lasted between 1000 and 1200 ms. The Stroop task included three blocks that each consisted of 72 trials (for a total of 216 trials). Before the formal task, participants completed one practice block, which consisted of 48 trials (50% congruent, 50% incongruent) that were similar to those used in the experimental blocks. Accuracy and RT feedback were provided following the completion of each trial in the practice block. There was no feedback during the experimental blocks.

### EEG/ERP recording and data processing

The EEG data were recorded at 64 scalp sites according to the 10–20 international placement system, with Ag/AgCl electrodes mounted in an elastic cap and a NeuroScan 4.5 EEG/ERP recording system (amplifier type: SynAmps 2). The left mastoid was used as the online reference. To monitor eye movements and blinks, horizontal and vertical electrooculograms (EOGs) were recorded with an additional four electrodes; two electrodes were placed above and below the left eye, and two electrodes were placed 1 cm lateral to the external canthus. The sampling rate was 1000 Hz, and an on-line band-pass filter of 0.01–100 Hz was used. The impedance of all electrodes was maintained below 5 kΩ.

The EEG data were processed offline with NeuroScan 4.5 software. The EEG data for all 64 electrodes were re-referenced to the average of the left and right mastoids. Ocular artifacts (blinks and eye movements) were removed from the EEG signal using a regression procedure implemented with the NeuroScan 4.5 software [52]. Epochs with artifacts that exceeded ±100 μV were excluded from further analysis. The averaged epochs for the ERP totaled 1000 ms, including 200 ms pre-stimuli as a baseline and 800 ms post-stimuli. The trials with correct responses were averaged in each condition.

The mean amplitudes of N2 and N450 were calculated at the negative waveform, which included time windows with ranges of 190–330 ms and 380–480 ms, respectively. The Fz, FCz, and Cz electrode points were chosen for the statistical analysis of both components. These time windows and sites were chosen to be consistent with previous reports in the literature[28], and a visual inspection of the grand-average waveforms.

### Data analysis

We used a 2 × 2 × 2 repeated-measures analyses of variance (ANOVA) with group (real rTMS and sham rTMS) × time (T1, at baseline, before stimulation; T2, immediately after 7 days of rTMS or sham rTMS;) × congruency (congruent stimuli or incongruent stimuli) factors for RTs and response accuracy. To analyze the Stroop interference effect (RT for incongruent stimuli minus RT for congruent stimuli), the ANOVA factors were group × time (2 × 2). Only the main effects of group, time, congruency, and the interactions related to group were reported because we focused on the effect of rTMS compared with sham rTMS. To analyze the mean amplitudes of the N2 and N450 components, the ANOVA factors were group, time, congruency, and electrode site (Fz, FCz, Cz; 2 × 2 × 2 × 3). The Greenhouse–Geisser correction was used to adjust for sphericity violations, and a post hoc comparison was applied to evaluate the significance of the interaction obtained in the ANOVA.

To clarify the behavioral significance of the N2 and N450 amplitudes, we performed a Pearson’s correlation analysis to explore the relationship between behavioral performances and the N2 and N450 amplitudes averaged across the three measurement sites both before and after stimulation in two conditions of the two groups respectively.

## Results

### Side effects of high-frequency rTMS

All participants completed the entire procedure for the present study. The participants were monitored during treatment for symptoms of discomfort or seizure. Throughout the study, the participants could stop participating in the study at any time and were encouraged to report if they experienced any discomfort associated with stimulation. Overall, rTMS was safe and well tolerated. One participant in the sham rTMS group noted minor scalp discomfort, and one participant in the rTMS group experienced a minor headache. No seizures or other adverse effects were observed.

### Behavioral data for the Stroop task

The RT data are presented in Table 1. In the RT analysis, no main group effect [F(1, 48) = 0.436, p = 0.512, η_p_^2^ = 0.009] was found. However, a significant main effect regarding time [F(1, 48) = 17.340, p<0.001, η_p_^2^ = 0.265] was found. The mean RTs decreased significantly at the second time point (T2, immediately after 7 days of rTMS or sham rTMS) compared with the first time point (T1, at baseline, before stimulation) (665.07ms vs. 708.87 ms, p<0.001). Additionally, a congruency effect was found [F(1, 48) = 134.138, p<0.001, η_p_^2^ = 0.736]. The mean RTs for the congruent stimuli were faster than those for the incongruent stimuli (616.4 ms vs. 756.8 ms, p<0.001). An interaction effect of time × group [F(1, 48) = 6.022, p = 0.018, η_p_^2^ = 0.211] was found. Further analysis revealed that the mean RT in the rTMS group was shorter than that in the sham rTMS group at T2 (p = 0.043), whereas there was no significant difference with respect to this parameter at T1 (p = 0.672). There were no significant congruency × group [F(1, 48) = 0.000, p = 0.999, η_p_^2^ = 0.000] or group × time × congruency [F(1, 48) = 0.063, p = 0.803, η_p_^2^ = 0.001] interactions.

**Table 1:** The mean RTs and standard deviations of the Stroop task at two time points in the rTMS and sham rTMS groups.

Group	RTs of the Stroop Task (ms)			
	Congruent		Incongruent	
	T1	T2	T1	T2
rTMS group	634.35±85.928	565.19±105.199 ^a^	794.41±132.17	724.35±112.029 ^a^
Sham rTMS group	621.38±64.331	607.82±83.158	785.38±137.598	762.90±141.004

T1, at baseline, before stimulation; T2, immediately after 7 days of rTMS or sham rTMS.

^a^ rTMS vs. sham rTMS: p<0.05.

For the Stroop interference effect, no main group effect [F(1, 48) = 0.094, p = 0.760, η_p_^2^ = 0.002] or group × time interactions[F(1, 48) = 0.063, p = 0.803, η_p_^2^ = 0.001] was found. The data of the Stroop interference effect are presented in Table 2.

**Table 2:** The Stroop interference effect at two time points in the rTMS and sham rTMS groups.

Group	The Stroop interference effect of the Stroop Task (ms)	
	T1	T2
rTMS group	160.06±135.150	159.164±61.585
Sham rTMS group	164.05±151.803	155.08±76.198

T1, at baseline, before stimulation; T2, immediately after 7 days of rTMS or sham rTMS.

The accuracy data are presented in Table 3. There was a significant main congruency effect [F(1, 48) = 103.002 p<0.001, η_p_^2^ = 0.682]. The accuracy of the response with the congruent stimuli was higher than that with the incongruent stimuli (98.47% vs. 94.09%, p<0.001). No other significant main effects or interactions were found.

**Table 3:** The mean accuracies and standard deviations of the Stroop task at two time points in the rTMS and sham rTMS groups.

Group	Accuracies of the Stroop Task (%)			
	Congruent		Incongruent	
	T1	T2	T1	T2
rTMS group	98.51±2.450 ^b^	98.33±1.559 ^b^	93.44±4.140	94.25±3.937
Sham rTMS group	98.70±1.535 ^b^	98.07±3.105 ^b^	94.85±4.089	93.78±5.926

T1, at baseline, before stimulation; T2, immediately after 7 days of rTMS or sham rTMS.

^b^ Congruent vs. Incongruent: p<0.001.

### ERP data

#### N2 component

**Fig 2:**
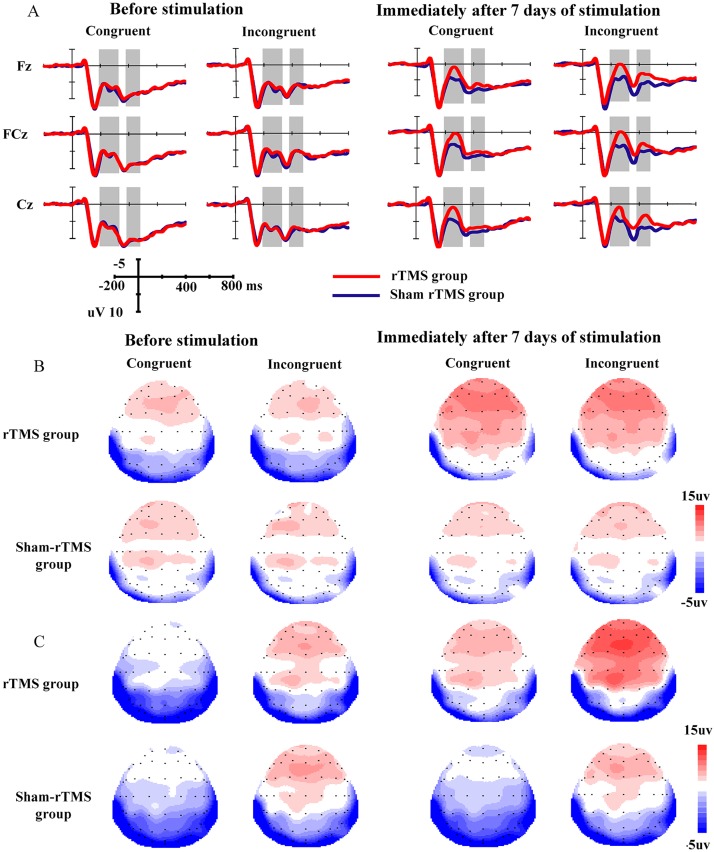
The grand-average N2 and N450 waveforms and their scalp distributions in the two groups. A: The grand-average N2 and N450 waveforms under two conditions (congruent, incongruent) at two time points (T1, T2) in the rTMS group and sham rTMS group. The gray areas represent the time windows of the measured mean amplitudes of N2 (190–330 ms) and N450 (380–480 ms). B: The scalp distributions of N2. C: The scalp distributions of N450.

About 8% of the trials were rejected after offline processing with NeuroScan 4.5 software. The grand-average N2 waveforms and their scalp distributions under two conditions (congruent, incongruent) at two time points (T1, T2) in the rTMS group and sham rTMS group are presented in Fig 2A and 2B. The results revealed a significant main effect of time [F(1, 48) = 76.191, p<0.001, η_p_^2^ = 0.613], i.e., the mean amplitude of the N2 component at T2 was more negative than that at T1. An interaction effect of time × group [F(1, 48) = 22.147, p<0.001, η_p_^2^ = 0.316] was found. Further analysis revealed that the mean amplitude of N2 in the rTMS group was larger than that in the sham rTMS group at T2 (p = 0.010), whereas there was no significant difference with respect to this parameter at T1 (p = 0.957). No significant main effects for congruency [F(1, 48) = 1.245, p = 0.270, η_p_^2^ = 0.025], group [F(1, 48) = 2.135, p = 0.150, η_p_^2^ = 0.043] or other interactions were found.

#### N450 component

The grand-average N450 waveforms and their scalp distributions under all conditions are presented in Fig 2A and 2C. A significant main congruency effect [F(1, 48) = 43.300, p<0.001, η_p_^2^ = 0.474] was found, i.e., the mean amplitude of the N450 component was more negative in response to incongruent stimuli than congruent stimuli. Additionally, a congruency × group interaction [F(1, 48) = 3.966, p = 0.050, η_p_^2^ = 0.076] was noted. Further analysis revealed that the mean amplitude of N450 in the rTMS group was larger than that in the sham rTMS group under the incongruent condition (p = 0.002) but not under the congruent condition (p = 0.965). Meanwhile, a significant main time effect [F(1, 48) = 8.041, p = 0.007, η_p_^2^ = 0.143] was found. The interaction effect of time × group [F(1, 48) = 4.575, p = 0.038, η_p_^2^ = 0.107] was significant. Further analysis revealed that the mean N450 amplitude in the rTMS group was larger than that in the sham rTMS group at T2 (p = 0.004) but not at T1 (p = 0.867). There were no main effects for group [F(1, 48) = 0.679, p = 0.414, η_p_^2^ = 0.014]. No other significant interaction effects were found.

### Correlation analysis

For the N2 component, the correlations under two conditions at two time points in the rTMS and sham rTMS groups are presented in Table 4. The mean amplitudes of N2 were negatively correlated with the RTs for the congruent stimuli and incongruent stimuli at two time points in two groups (p<0.05), indicating that the larger the N2 amplitude, the shorter the RT for both types of stimuli. However, the correlation was not enhanced or diminished by rTMS. The mean amplitude of N2 was not significantly correlated with accuracy for the congruent stimuli or the incongruent stimuli at two time points in the rTMS and sham rTMS groups (p>0.05).

**Table 4:** The correlations between N2 amplitudes and RTs under two conditions at two time points in the rTMS and sham rTMS groups.

Group	Correlations between N2 amplitudes and RTs (correlation coefficient, r value)			
	Congruent		Incongruent	
	T1	T2	T1	T2
rTMS group	-0.334 ^c^	-0.370 ^c^	-0.331 ^c^	-0.416 ^c^
Sham rTMS group	-0.302 ^c^	-0.313 ^c^	-0.370 ^c^	-0.307 ^c^

T1, at baseline, before stimulation; T2, immediately after 7 days of rTMS or sham rTMS.

^c^ Pearson’s correlation analysis: p<0.05; r value: ±0.3-±0.5: low correlation; ±0.5-±0.8: moderate correlation;±0.8-±1: high correlation.

For the N450 component, the correlations under two conditions at two time points in the rTMS and sham rTMS groups are presented in Table 5. The mean amplitudes of N450 were negatively correlated with the RTs for the incongruent stimuli at two time points in two groups (p<0.05). Furthermore, the correlation was enhanced significantly by rTMS (r value: -0.616 vs -0.381) rather than sham rTMS. However, the mean amplitude of N450 was not correlated with RTs for the congruent stimuli and accuracy for the congruent stimuli or incongruent stimuli (p>0.05).

**Table 5:** The correlations between N450 amplitudes and RTs under two conditions at two time points in the rTMS and sham rTMS groups.

Group	Correlations between N450 and RTs (correlation coefficient, r value)			
	Congruent		Incongruent	
	T1	T2	T1	T2
rTMS group	-0.089	-0.090	-0.381 ^d^	-0.616 ^d^
Sham rTMS group	-0.103	-0.077	-0.365 ^d^	-0.302 ^d^

T1, at baseline, before stimulation; T2, immediately after 7 days of rTMS or sham rTMS.

^d^ Pearson’s correlation analysis: p<0.05; r value: ±0.3-±0.5: low correlation; ±0.5-±0.8: moderate correlation; >±0.8-±1: high correlation.

## Discussion

The present study first focused on the effects of multiple sessions of high-frequency rTMS (10 Hz) applied over the left DLPFC on cognitive control in healthy young participants. The main results can be summarized as follows. As expected, there were significant behavioral improvements of the Stroop task in the rTMS group, i.e., the RTs on both the incongruent and congruent trials decreased significantly after real rTMS. The frontocentral N2 and N450 amplitudes in the rTMS group were larger than those in the sham rTMS group at T2, whereas there was no significant difference at T1. In addition, the amplitudes of N450 were larger in the rTMS group than in the sham rTMS group for incongruent stimuli but not congruent stimuli. The negative correlations between the mean amplitudes of both N2 and N450 and the RTs were found in the current study, however, the latter correlations were restricted to incongruent trials.

Behaviorally, the classical Stroop interference effect was successfully obtained in the present study, in which longer RTs and decreased accuracies occurred in the incongruent trials compared with the congruent trials. This result suggested that the Stroop paradigm that was applied in our study was very reliable. We found that rTMS over the left DLPFC decreased the RTs of both congruent and incongruent trials in the Stroop task. Our result was consistent with that of a previous study[13]. Vanderhasselt MA [13] found that the high-frequency (10 Hz) rTMS over the left DLPFC decreased the RTs of both congruent and incongruent trials in a Stroop task in healthy females. Evidence from PET and fMRI studies has shown that the neural circuitry of the left DLPFC-cingulate cortex is the anatomical basis of cognitive control in a Stroop task in healthy subjects [53, 54]. It was worth noting that there was no Stroop interference effect of rTMS. That is, there was no differential effect of rTMS on RTs for incongruent vs. congruent trials. Consequently, an alternative explanation is a “floor effect” [55] such that rTMS is unable to produce an additional reduction in the RTs for incongruent trials when the RTs for incongruent trials have been dramatically reduced in the rTMS group. Our results that HF-rTMS over the left DLPFC decreased the RTs of both congruent and incongruent trials in the Stroop task suggested that HF-rTMS enhance psychomotor speed in healthy participants. Previous clinical studies showed a positive impact of HF-rTMS treatment on psychomotor symptoms by targeting the left DLPFC in patients with major depression [56, 57]. We first revealed that multiple sessions of high frequency rTMS over the left DLPFC had a positive effect on Stroop task in young healthy participants. This result encourages us to perform further research on the impact of rTMS on various cognitive functions of healthy people. That is, high-frequency rTMS may be a promising and potential technique to enable healthy people to achieve better performance in various cognitive tasks.

It should be noted that neutral stimuli (i.e., semantically meaningless stimuli) were not used in the Stroop task in our study. According to previous literatures, the common finding of Stroop effect is that RT to an incongruent color word is slower than to a neutral stimulus [58, 59]. And RT to a congruent color word is faster than or similar to the RT for the neutral condition [60] in the Stroop task which neutral stimuli were involved. Whereas the interference effect (The RTs for the incongruent stimuli minus the RTs for the neutral stimuli) is large and robust, the facilitation effect (difference between the RTs for the neutral stimuli and the RTs for the congruent stimuli) is small and fragile. That is, with respect to the informational conflict, the neutral condition is more conflicting than the congruent condition. It means also the information produced by the congruent stimuli is more compatible than that produced by any kind of neutral stimuli[61]. In addition, consulting the previous literatures about effects of rTMS, or other intervention, on the Stroop task, we found that classical Stroop color-word task (neutral stimuli were not involved) was usually adopted and the outcomes have been robust [13, 17, 62]. These results suggest that the classical color-word Stroop task is a very reliable paradigm. Therefore, we chose the classical color-word Stroop task (neutral stimuli were not involved) in our study. We will try to use other Stroop tasks (e.g., neutral stimuli were involved or modified Stroop tasks) to reveal more relevant psychological mechanism of rTMS effects in the future study.

The data indicated that the frontocentral N2 amplitudes were greater under both congruent and incongruent conditions for participants who received 7 days of rTMS treatment over the left DLPFC compared with those who received the sham rTMS treatment; there was no difference in this parameter before treatment. According to previous studies, N2 may represent the strategic deployment of cognitive control [34, 35] during a given task. N2 amplitudes are sensitive to manipulations of stimuli probability and are largest for highly probable stimuli but not with incongruent stimuli[36]. In the current paradigm, in which there was an equal probability of congruent and incongruent trials, the N2 amplitudes were similar between the incongruent and congruent stimuli. Our results regarding the N2 amplitudes suggest that 7 days of rTMS treatment over the left DLPFC can significantly increase neural activity at the strategic deployment of cognitive control stage in a task that involves competing responses.

For the frontocentral N450 component, which is clearly distinct from and later than the frontocentral N2, our results demonstrated that the amplitudes were greater in the rTMS group compared with the sham rTMS group. Previous studies have suggested that N450 represents a mechanism for conflict monitoring that is sensitive to incongruent compared with congruent stimuli in tasks that involve cognitive control[30]. Our results suggest that 7 days of rTMS over the left DLPFC may significantly increase neural activity at the conflict-monitoring stage of cognitive control processing during the Stroop task. We also found that the N450 amplitudes were enhanced by rTMS under the incongruent condition during the Stroop task but not under the congruent condition. In both the current study and previous studies, the N450 component demonstrated enhanced amplitudes in response to incongruent stimuli [63, 64], which requires a greater recruitment of cognitive resources [28, 65] from the ACC (a conflict evaluator) and DLPFC (a cognitive control implementer) [30]. Our results suggest that rTMS had a greater effect on the neural circuitry of the left DLPFC-cingulate cortex in situations during which additional cognitive resources are required (e.g., incongruent stimuli) than situations in which fewer cognitive resources are necessary.

The current findings indicate that the amplitudes of both the N2 and N450 components were significantly increased by 7 days of rTMS over the left DLPFC. These two components reflect different stages of cognitive control processing. However, the interactions and distinctions between N2 and N450 remain subtle because these two components are rarely compared within the same paradigm. Based on previous reports in the literature, we supposed that the N2 component reflects the strategic deployment of cognitive control [33, 34], whereas N450 reflects conflict-monitoring processing of cognitive control and is sensitive to higher conflict-interference conditions [30]. Larson [30] proposed that larger N2 and N450 amplitudes are related to an improved cognitive control function and greater ACC activity, which recruits more neural resources from the prefrontal cortex, especially the DLPFC, during a given task [30]. Previous studies have suggested that applying rTMS over the prefrontal cortex can affect target discrimination-related P3 [25] and error monitoring related ERN [40]. Our results extended these previous results by suggesting that high-frequency rTMS application could have positive effects on multiple stages of cognitive control processing from the point of electrophysiology and decreased RTs in both congruent and incongruent conditions of the Stroop task behaviorally in young healthy participants.

However, it is debatable whether enhanced N2 and N450 amplitudes reflect an increased resource recruitment of cognitive control or an increased effeciency of resources to deploy for conflict resolution. According to previous studies, there is already growing evidence that better behavioral performance in attentional and executive control tasks is associated with more pronounced ERP components [66]. For example, larger N2 and N450 [67, 68] and better behavioral performance were obtained in young compared to old participants. Long-term physical activity will enhance the amplitudes of the frontocentral N2 and N450 components in a Stroop task in aging people [66]. The results suggest that long-term physical activity may generally enhance activity in the frontal cortex which recruits more efficient resources. In addition, a large number of neuropsychiatric disorders that exhibit deficient cognitive control functions elicited reduced N2 and N450 amplitudes. Compared to healthy controls, Major Depressive Disorder (MDD)[69], attention-deficit-hyperactivity disorder (AHDH)[70] and schizophrenia [71] patients showed reduced N2 amplitudes and attenuated N450 amplitudes. Evidence from neuroimaging studies indicates that depressive symptomatology is associated with the inefficient recruitment of prefrontal brain regions while performing Stroop tasks [72] Overall, N2 and N450 showed decreased amplitudes in aging people and patients with a number of neuropsychiatric disorders who had deficits in cognitive control. However, effective interventions and treatment of these people can improve behavioral performance and increase the amplitudes of N2 and N450. In general, the ERP amplitude reflects the amount of resources employed in a given task, thus providing information about differing amounts of mental effort required for various stimuli. Hence, increased amplitudes of N2 and N450 accompanied with better behavioral performance are associated with the recruitment of more neural resources in the prefrontal brain regions. Furthermore, the negative correlations between the mean amplitudes of both N2 and N450 and the RTs in the current study, i.e., the larger the amplitudes of N2 and N450, the shorter the RTs, also support the view that the enhanced amplitudes of N2 and N450 are associated with better task performance. We also found that the correlation between N450 amplitudes and RTs for incongruent stimuli was enhanced by rTMS significantly. This indicates that rTMS can enhance efficiency of resources to deploy for conflict resolution. That is, the ERP data provide a basis for commenting about whether rTMS can enhance availability or efficiency of cognitive resource allocation. In our study, we found that multiple sessions of HF-rTMS can decrease the RTs and increase the amplitudes of both N2 and N450 compared with sham rTMS. And the correlation between N450 amplitudes and RTs for incongruent stimuli was enhanced significantly by rTMS. Therefore, we suggested that high-frequency rTMS over the left DLPFC not only recruits more neural resources from the prefrontal cortex by inducing an electrophysiologically excitatory effect but also enhances efficiency of resources to deploy for conflict resolution in healthy young people.

The current study has certain limitations that must be addressed. First, we found that rTMS over the left DLPFC elicited larger frontocentral N2 and N450 amplitudes; however, the effects of rTMS on local blood flow and metabolic changes remain unclear. The combination of rTMS with other neuroimaging methods, such as functional magnetic resonance imaging (fMRI), magnetic resonance spectroscopy (MRS) or positron emission tomography (PET), could provide fruitful avenues for research. Second, the generalizability of the current findings may also be limited by our utilization of a relatively small sample of young adults. Therefore, additional studies with larger samples are warranted. Third, in our study, the conventional “5 cm rule” (the method of Pascual-Leone) was applied to target the left DLPFC. This conventional method is not entirely accurate compared with MRI-guided neuronavigation, which can target a desired cortical region directly [73]. However, many previous studies have used this technique and the outcomes have been robust and reproducible [74]. Fourth, there was no significant difference in Stroop interference effects between the two groups, further studies and different Stroop paradigms (e.g., the involvement of neutral stimuli or a different probability of congruent and incongruent trials) are needed to clarify the mechanism of HF-rTMS. Fifth, our study focused on the cumulative effects of rTMS; however, the long-term effects and the duration of the positive effects of rTMS are unclear.

## Conclusions

In conclusion, the results of the current study suggest that multiple sessions of high-frequency rTMS over the left DLPFC can decrease RTs under both congruent and incongruent conditions, increase neural activity in the prefrontal areas by inducing an electrophysiologically excitatory effect and enhance efficiency of resources to deploy for conflict resolution during multiple stages of cognitive control processing in healthy young people. Further research is required to expand rTMS applications by involving more diverse groups of participants and investigating the long-term effects of rTMS.

## Supporting information

S1 Table The mean N2 amplitudes of the Stroop task under two conditions at two time points in the rTMS and sham rTMS groups. (PDF)

S2 Table The mean N450 amplitudes of the Stroop task under two conditions at two time points in the rTMS and sham rTMS groups. (PDF)

## References

[1] Barker AT. An introduction to the basic principles of magnetic nerve stimulation. Journal of clinical neurophysiology: official publication of the American Electroencephalographic Society. 1991;8(1):26–37. . 2019648 10.1097/00004691-199101000-00005

[2] Haraldsson HM, Ferrarelli F, Kalin NH, Tononi G. Transcranial Magnetic Stimulation in the investigation and treatment of schizophrenia: a review. Schizophrenia research. 2004;71(1):1–16. doi: 10.1016/j.schres.2003.10.006 . 15374567 10.1016/j.schres.2003.10.006

[3] Rossi S, Rossini PM. TMS in cognitive plasticity and the potential for rehabilitation. Trends in cognitive sciences. 2004;8(6):273–9. doi: 10.1016/j.tics.2004.04.012 . 15165553 10.1016/j.tics.2004.04.012

[4] Fox P, Ingham R, George MS, Mayberg H, Ingham J, Roby J, et al Imaging human intra-cerebral connectivity by PET during TMS. Neuroreport. 1997;8(12):2787–91. . 9295118 10.1097/00001756-199708180-00027

[5] Eliasova I, Anderkova L, Marecek R, Rektorova I. Non-invasive brain stimulation of the right inferior frontal gyrus may improve attention in early Alzheimer's disease: a pilot study. Journal of the neurological sciences. 2014;346(1–2):318–22. doi: 10.1016/j.jns.2014.08.036 . 25216556 10.1016/j.jns.2014.08.036

[6] Little JT, Kimbrell TA, Wassermann EM, Grafman J, Figueras S, Dunn RT, et al Cognitive effects of 1- and 20-hertz repetitive transcranial magnetic stimulation in depression: preliminary report. Neuropsychiatry, neuropsychology, and behavioral neurology. 2000;13(2):119–24. . 10780630

[7] Padberg F, Zwanzger P, Thoma H, Kathmann N, Haag C, Greenberg BD, et al Repetitive transcranial magnetic stimulation (rTMS) in pharmacotherapy-refractory major depression: comparative study of fast, slow and sham rTMS. Psychiatry research. 1999;88(3):163–71. . 10622338 10.1016/s0165-1781(99)00092-x

[8] Sole-Padulles C, Bartres-Faz D, Junque C, Clemente IC, Molinuevo JL, Bargallo N, et al Repetitive transcranial magnetic stimulation effects on brain function and cognition among elders with memory dysfunction. A randomized sham-controlled study. Cerebral cortex. 2006;16(10):1487–93. doi: 10.1093/cercor/bhj083 . 16339086 10.1093/cercor/bhj083

[9] Rektorova I, Megova S, Bares M, Rektor I. Cognitive functioning after repetitive transcranial magnetic stimulation in patients with cerebrovascular disease without dementia: a pilot study of seven patients. Journal of the neurological sciences. 2005;229–230:157–61. doi: 10.1016/j.jns.2004.11.021 . 15760635 10.1016/j.jns.2004.11.021

[10] Cotelli M, Calabria M, Manenti R, Rosini S, Zanetti O, Cappa SF, et al Improved language performance in Alzheimer disease following brain stimulation. Journal of neurology, neurosurgery, and psychiatry. 2011;82(7):794–7. doi: 10.1136/jnnp.2009.197848 . 20574108 10.1136/jnnp.2009.197848

[11] Martis B, Alam D, Dowd SM, Hill SK, Sharma RP, Rosen C, et al Neurocognitive effects of repetitive transcranial magnetic stimulation in severe major depression. Clinical neurophysiology: official journal of the International Federation of Clinical Neurophysiology. 2003;114(6):1125–32. . 12804681 10.1016/s1388-2457(03)00046-4

[12] Ahmed MA, Darwish ES, Khedr EM, El Serogy YM, Ali AM. Effects of low versus high frequencies of repetitive transcranial magnetic stimulation on cognitive function and cortical excitability in Alzheimer's dementia. Journal of neurology. 2012;259(1):83–92. doi: 10.1007/s00415-011-6128-4 . 21671144 10.1007/s00415-011-6128-4

[13] Vanderhasselt MA, De Raedt R, Baeken C, Leyman L, D'Haenen H. The influence of rTMS over the left dorsolateral prefrontal cortex on Stroop task performance. Experimental brain research. 2006;169(2):279–82. doi: 10.1007/s00221-005-0344-z . 16418843 10.1007/s00221-005-0344-z

[14] Hwang JH, Kim SH, Park CS, Bang SA, Kim SE. Acute high-frequency rTMS of the left dorsolateral prefrontal cortex and attentional control in healthy young men. Brain research. 2010;1329:152–8. doi: 10.1016/j.brainres.2010.03.013 . 20226772 10.1016/j.brainres.2010.03.013

[15] Rounis E, Stephan KE, Lee L, Siebner HR, Pesenti A, Friston KJ, et al Acute changes in frontoparietal activity after repetitive transcranial magnetic stimulation over the dorsolateral prefrontal cortex in a cued reaction time task. The Journal of neuroscience: the official journal of the Society for Neuroscience. 2006;26(38):9629–38. doi: 10.1523/JNEUROSCI.2657-06.2006 . 16988033 10.1523/JNEUROSCI.2657-06.2006 PMC6674444

[16] Eisenegger C, Treyer V, Fehr E, Knoch D. Time-course of "off-line" prefrontal rTMS effects—a PET study. NeuroImage. 2008;42(1):379–84. doi: 10.1016/j.neuroimage.2008.04.172 . 18511301 10.1016/j.neuroimage.2008.04.172

[17] Kim SH, Han HJ, Ahn HM, Kim SA, Kim SE. Effects of five daily high-frequency rTMS on Stroop task performance in aging individuals. Neuroscience research. 2012;74(3–4):256–60. doi: 10.1016/j.neures.2012.08.008 . 22974554 10.1016/j.neures.2012.08.008

[18] Takahashi S, Ukai S, Tsuji T, Ueyama T, Kono M, Yamanaka N, et al Reduction of cortical excitability and increase of thalamic activity in a low-frequency rTMS treatment for chronic tinnitus. Neurocase. 2015;21(3):339–44. doi: 10.1080/13554794.2014.893000 . 24606019 10.1080/13554794.2014.893000

[19] Baeken C, Marinazzo D, Everaert H, Wu GR, Van Hove C, Audenaert K, et al The Impact of Accelerated HF-rTMS on the Subgenual Anterior Cingulate Cortex in Refractory Unipolar Major Depression: Insights From 18FDG PET Brain Imaging. Brain stimulation. 2015;8(4):808–15. doi: 10.1016/j.brs.2015.01.415 . 25744500 10.1016/j.brs.2015.01.415

[20] Dall'Agnol L, Medeiros LF, Torres IL, Deitos A, Brietzke A, Laste G, et al Repetitive transcranial magnetic stimulation increases the corticospinal inhibition and the brain-derived neurotrophic factor in chronic myofascial pain syndrome: an explanatory double-blinded, randomized, sham-controlled trial. The journal of pain: official journal of the American Pain Society. 2014;15(8):845–55. doi: 10.1016/j.jpain.2014.05.001 . 24865417 10.1016/j.jpain.2014.05.001

[21] Yang HY, Liu Y, Xie JC, Liu NN, Tian X. Effects of repetitive transcranial magnetic stimulation on synaptic plasticity and apoptosis in vascular dementia rats. Behavioural brain research. 2015;281:149–55. doi: 10.1016/j.bbr.2014.12.037 . 25541037 10.1016/j.bbr.2014.12.037

[22] Esslinger C, Schuler N, Sauer C, Gass D, Mier D, Braun U, et al Induction and quantification of prefrontal cortical network plasticity using 5 Hz rTMS and fMRI. Human brain mapping. 2014;35(1):140–51. doi: 10.1002/hbm.22165 . 22965696 10.1002/hbm.22165 PMC6868951

[23] van den Heuvel OA, Van Gorsel HC, Veltman DJ, Van Der Werf YD. Impairment of executive performance after transcranial magnetic modulation of the left dorsal frontal-striatal circuit. Human brain mapping. 2013;34(2):347–55. doi: 10.1002/hbm.21443 . 22076808 10.1002/hbm.21443 PMC6870447

[24] De Raedt R, Leyman L, Baeken C, Van Schuerbeek P, Luypaert R, Vanderhasselt MA, et al Neurocognitive effects of HF-rTMS over the dorsolateral prefrontal cortex on the attentional processing of emotional information in healthy women: an event-related fMRI study. Biological psychology. 2010;85(3):487–95. doi: 10.1016/j.biopsycho.2010.09.015 . 20923694 10.1016/j.biopsycho.2010.09.015

[25] Evers S, Bockermann I, Nyhuis PW. The impact of transcranial magnetic stimulation on cognitive processing: an event-related potential study. Neuroreport. 2001;12(13):2915–8. . 11588602 10.1097/00001756-200109170-00032

[26] Rektor I, Balaz M, Bockova M. Cognitive event-related potentials and oscillations in the subthalamic nucleus. Neuro-degenerative diseases. 2010;7(1–3):160–2. doi: 10.1159/000289228 . 20197697 10.1159/000289228

[27] Millner AJ, Jaroszewski AC, Chamarthi H, Pizzagalli DA. Behavioral and electrophysiological correlates of training-induced cognitive control improvements. NeuroImage. 2012;63(2):742–53. doi: 10.1016/j.neuroimage.2012.07.032 . 22836178 10.1016/j.neuroimage.2012.07.032 PMC3601637

[28] Carter CS, van Veen V. Anterior cingulate cortex and conflict detection: an update of theory and data. Cognitive, affective & behavioral neuroscience. 2007;7(4):367–79. . 18189010 10.3758/cabn.7.4.367

[29] Kerns JG. Anterior cingulate and prefrontal cortex activity in an FMRI study of trial-to-trial adjustments on the Simon task. NeuroImage. 2006;33(1):399–405. doi: 10.1016/j.neuroimage.2006.06.012 . 16876434 10.1016/j.neuroimage.2006.06.012

[30] Larson MJ, Clayson PE, Clawson A. Making sense of all the conflict: a theoretical review and critique of conflict-related ERPs. International journal of psychophysiology: official journal of the International Organization of Psychophysiology. 2014;93(3):283–97. doi: 10.1016/j.ijpsycho.2014.06.007 . 24950132 10.1016/j.ijpsycho.2014.06.007

[31] Folstein JR, Van Petten C. Influence of cognitive control and mismatch on the N2 component of the ERP: a review. Psychophysiology. 2008;45(1):152–70. doi: 10.1111/j.1469-8986.2007.00602.x . 17850238 10.1111/j.1469-8986.2007.00602.x PMC2365910

[32] Nieuwenhuis S, Yeung N, Cohen JD. Stimulus modality, perceptual overlap, and the go/no-go N2. Psychophysiology. 2004;41(1):157–60. doi: 10.1046/j.1469-8986.2003.00128.x . 14693011 10.1046/j.1469-8986.2003.00128.x

[33] van Veen V, Carter CS. The anterior cingulate as a conflict monitor: fMRI and ERP studies. Physiology & behavior. 2002;77(4–5):477–82. . 12526986 10.1016/s0031-9384(02)00930-7

[34] Gratton G, Coles MG, Donchin E. Optimizing the use of information: strategic control of activation of responses. Journal of experimental psychology General. 1992;121(4):480–506. . 1431740 10.1037//0096-3445.121.4.480

[35] Gratton G, Coles MG, Sirevaag EJ, Eriksen CW, Donchin E. Pre- and poststimulus activation of response channels: a psychophysiological analysis. Journal of experimental psychology Human perception and performance. 1988;14(3):331–44. . 2971764 10.1037//0096-1523.14.3.331

[36] Bartholow BD, Pearson MA, Dickter CL, Sher KJ, Fabiani M, Gratton G. Strategic control and medial frontal negativity: beyond errors and response conflict. Psychophysiology. 2005;42(1):33–42. doi: 10.1111/j.1469-8986.2005.00258.x . 15720579 10.1111/j.1469-8986.2005.00258.x

[37] Yeung N, Nieuwenhuis S. Dissociating response conflict and error likelihood in anterior cingulate cortex. The Journal of neuroscience: the official journal of the Society for Neuroscience. 2009;29(46):14506–10. doi: 10.1523/JNEUROSCI.3615-09.2009 . 19923284 10.1523/JNEUROSCI.3615-09.2009 PMC2831178

[38] Liotti M, Woldorff MG, Perez R, Mayberg HS. An ERP study of the temporal course of the Stroop color-word interference effect. Neuropsychologia. 2000;38(5):701–11. . 10689046 10.1016/s0028-3932(99)00106-2

[39] Markela-Lerenc J, Ille N, Kaiser S, Fiedler P, Mundt C, Weisbrod M. Prefrontal-cingulate activation during executive control: which comes first? Brain research Cognitive brain research. 2004;18(3):278–87. . 14741314 10.1016/j.cogbrainres.2003.10.013

[40] Rollnik JD, Schroder C, Rodriguez-Fornells A, Kurzbuch AR, Dauper J, Moller J, et al Functional lesions and human action monitoring: combining repetitive transcranial magnetic stimulation and event-related brain potentials. Clinical neurophysiology: official journal of the International Federation of Clinical Neurophysiology. 2004;115(1):145–53. . 14706482 10.1016/j.clinph.2003.05.001

[41] Van den Eynde F, Claudino AM, Campbell IC, Schmidt U. Immediate cognitive effects of repetitive Transcranial Magnetic Stimulation in eating disorders: a pilot study. Eating and weight disorders: EWD. 2011;16(1):e45–8. . 21727781 10.1007/BF03327520

[42] Huang CC, Su TP, Shan IK, Wei IH. Effect of 5 Hz repetitive transcranial magnetic stimulation on cognition during a Go/NoGo task. Journal of psychiatric research. 2004;38(5):513–20. doi: 10.1016/j.jpsychires.2004.01.006 . 15380402 10.1016/j.jpsychires.2004.01.006

[43] Guse B, Falkai P, Wobrock T. Cognitive effects of high-frequency repetitive transcranial magnetic stimulation: a systematic review. Journal of neural transmission. 2010;117(1):105–22. doi: 10.1007/s00702-009-0333-7 . 19859782 10.1007/s00702-009-0333-7 PMC3085788

[44] Hamilton M. Development of a Psychiatric Rating Scale for Primary Depression. Brit J Soc Clin Psychol. 1967;6:278–96. 6080235 10.1111/j.2044-8260.1967.tb00530.x

[45] Hamilton M. The assessment of anxiety by rating scale. Brit J Med Psychol. 1959;32:50–5. 13638508 10.1111/j.2044-8341.1959.tb00467.x

[46] Folstein MF, Folstein SE, McHugh PR. "Mini-mental state". A practical method for grading the cognitive state of patients for the clinician. Journal of psychiatric research. 1975;12(3):189–98. . 1202204 10.1016/0022-3956(75)90026-6

[47] Nasreddine ZS, Phillips NA, Bedirian V, Charbonneau S, Whitehead V, Collin I, et al The Montreal Cognitive Assessment, MoCA: a brief screening tool for mild cognitive impairment. Journal of the American Geriatrics Society. 2005;53(4):695–9. doi: 10.1111/j.1532-5415.2005.53221.x . 15817019 10.1111/j.1532-5415.2005.53221.x

[48] Wassermann EM. Risk and safety of repetitive transcranial magnetic stimulation: report and suggested guidelines from the International Workshop on the Safety of Repetitive Transcranial Magnetic Stimulation, June 5–7, 1996. Electroencephalography and clinical neurophysiology. 1998;108(1):1–16. . 9474057 10.1016/s0168-5597(97)00096-8

[49] Anand S, Hotson J. Transcranial magnetic stimulation: neurophysiological applications and safety. Brain and cognition. 2002;50(3):366–86. . 12480484 10.1016/s0278-2626(02)00512-2

[50] Ren C, Tarjan PP, Popovic DB. A novel electric design for electromagnetic stimulation—the Slinky coil. IEEE transactions on bio-medical engineering. 1995;42(9):918–25. doi: 10.1109/10.412658 . 7558066 10.1109/10.412658

[51] Pascual-Leone A, Rubio B, Pallardo F, Catala MD. Rapid-rate transcranial magnetic stimulation of left dorsolateral prefrontal cortex in drug-resistant depression. Lancet. 1996;348(9022):233–7. . 8684201 10.1016/s0140-6736(96)01219-6

[52] Semlitsch HV, Anderer P, Schuster P, Presslich O. A solution for reliable and valid reduction of ocular artifacts, applied to the P300 ERP. Psychophysiology. 1986;23(6):695–703. . 3823345 10.1111/j.1469-8986.1986.tb00696.x

[53] Harrison BJ, Shaw M, Yucel M, Purcell R, Brewer WJ, Strother SC, et al Functional connectivity during Stroop task performance. NeuroImage. 2005;24(1):181–91. doi: 10.1016/j.neuroimage.2004.08.033 . 15588609 10.1016/j.neuroimage.2004.08.033

[54] MacDonald AW 3rd, Cohen JD, Stenger VA, Carter CS. Dissociating the role of the dorsolateral prefrontal and anterior cingulate cortex in cognitive control. Science. 2000;288(5472):1835–8. . 10846167 10.1126/science.288.5472.1835

[55] Scherr M, Kunz A, Doll A, Mutzenbach JS, Broussalis E, Bergmann HJ, et al Ignoring floor and ceiling effects may underestimate the effect of carotid artery stenting on cognitive performance. Journal of neurointerventional surgery. 2016;8(7):747–51. doi: 10.1136/neurintsurg-2014-011612 . 26063796 10.1136/neurintsurg-2014-011612

[56] Hoeppner J, Padberg F, Domes G, Zinke A, Herpertz SC, Grossheinrich N, et al Influence of repetitive transcranial magnetic stimulation on psychomotor symptoms in major depression. European archives of psychiatry and clinical neuroscience. 2010;260(3):197–202. doi: 10.1007/s00406-009-0039-8 . 19680706 10.1007/s00406-009-0039-8

[57] Baeken C, De Raedt R, Santermans L, Zeeuws D, Vanderhasselt MA, Meers M, et al HF-rTMS treatment decreases psychomotor retardation in medication-resistant melancholic depression. Progress in neuro-psychopharmacology & biological psychiatry. 2010;34(4):684–7. doi: 10.1016/j.pnpbp.2010.03.021 . 20307619 10.1016/j.pnpbp.2010.03.021

[58] MacLeod CM. Half a century of research on the Stroop effect: an integrative review. Psychological bulletin. 1991;109(2):163–203. . 2034749 10.1037/0033-2909.109.2.163

[59] Stroop J. Studies in interference in serial verbal reactions. J Exp Psychol Gen. 1935;18:643–61.

[60] Dalrymple-Alford EC, Budayer B. Examination of some aspects of the Stroop Color-Word Test. Perceptual and motor skills. 1966;23(3):1211–4. doi: 10.2466/pms.1966.23.3f.1211 . 5972923 10.2466/pms.1966.23.3f.1211

[61] Goldfarb L, Henik A. Evidence for task conflict in the Stroop effect. Journal of experimental psychology Human perception and performance. 2007;33(5):1170–6. doi: 10.1037/0096-1523.33.5.1170 . 17924815 10.1037/0096-1523.33.5.1170

[62] Vanderhasselt MA, De Raedt R, Baeken C, Leyman L, Clerinx P, D'Haenen H. The influence of rTMS over the right dorsolateral prefrontal cortex on top-down attentional processes. Brain research. 2007;1137(1):111–6. doi: 10.1016/j.brainres.2006.12.050 . 17229406 10.1016/j.brainres.2006.12.050

[63] West R, Alain C. Effects of task context and fluctuations of attention on neural activity supporting performance of the stroop task. Brain research. 2000;873(1):102–11. . 10915815 10.1016/s0006-8993(00)02530-0

[64] Chouiter L, Dieguez S, Annoni JM, Spierer L. High and low stimulus-driven conflict engage segregated brain networks, not quantitatively different resources. Brain topography. 2014;27(2):279–92. doi: 10.1007/s10548-013-0303-0 . 23813270 10.1007/s10548-013-0303-0

[65] Botvinick MM, Cohen JD, Carter CS. Conflict monitoring and anterior cingulate cortex: an update. Trends in cognitive sciences. 2004;8(12):539–46. doi: 10.1016/j.tics.2004.10.003 . 15556023 10.1016/j.tics.2004.10.003

[66] Gajewski PD, Falkenstein M. Long-term habitual physical activity is associated with lower distractibility in a Stroop interference task in aging: Behavioral and ERP evidence. Brain and cognition. 2015;98:87–101. doi: 10.1016/j.bandc.2015.06.004 . 26160263 10.1016/j.bandc.2015.06.004

[67] Lucci G, Berchicci M, Spinelli D, Taddei F, Di Russo F. The effects of aging on conflict detection. PloS one. 2013;8(2):e56566 doi: 10.1371/journal.pone.0056566 . 23418584 10.1371/journal.pone.0056566 PMC3572012

[68] West R. The effects of aging on controlled attention and conflict processing in the Stroop task. Journal of cognitive neuroscience. 2004;16(1):103–13. doi: 10.1162/089892904322755593 . 15006040 10.1162/089892904322755593

[69] Holmes AJ, Pizzagalli DA. Response conflict and frontocingulate dysfunction in unmedicated participants with major depression. Neuropsychologia. 2008;46(12):2904–13. doi: 10.1016/j.neuropsychologia.2008.05.028 . 18577391 10.1016/j.neuropsychologia.2008.05.028 PMC2538441

[70] Pliszka SR, Liotti M, Woldorff MG. Inhibitory control in children with attention-deficit/hyperactivity disorder: event-related potentials identify the processing component and timing of an impaired right-frontal response-inhibition mechanism. Biological psychiatry. 2000;48(3):238–46. . 10924667 10.1016/s0006-3223(00)00890-8

[71] Minzenberg MJ, Gomes GC, Yoon JH, Swaab TY, Carter CS. Disrupted action monitoring in recent-onset psychosis patients with schizophrenia and bipolar disorder. Psychiatry research. 2014;221(1):114–21. doi: 10.1016/j.pscychresns.2013.11.003 . 24314907 10.1016/j.pscychresns.2013.11.003 PMC4019327

[72] Krompinger JW, Simons RF. Cognitive inefficiency in depressive undergraduates: stroop processing and ERPs. Biological psychology. 2011;86(3):239–46. doi: 10.1016/j.biopsycho.2010.12.004 . 21185350 10.1016/j.biopsycho.2010.12.004

[73] Mir-Moghtadaei A, Caballero R, Fried P, Fox MD, Lee K, Giacobbe P, et al Concordance Between BeamF3 and MRI-neuronavigated Target Sites for Repetitive Transcranial Magnetic Stimulation of the Left Dorsolateral Prefrontal Cortex. Brain stimulation. 2015;8(5):965–73. doi: 10.1016/j.brs.2015.05.008 . 26115776 10.1016/j.brs.2015.05.008 PMC4833442

[74] Haghighi M, Shayganfard M, Jahangard L, Ahmadpanah M, Bajoghli H, Pirdehghan A, et al Repetitive Transcranial Magnetic Stimulation (rTMS) improves symptoms and reduces clinical illness in patients suffering from OCD—Results from a single-blind, randomized clinical trial with sham cross-over condition. Journal of psychiatric research. 2015;68:238–44. doi: 10.1016/j.jpsychires.2015.06.020 . 26228425 10.1016/j.jpsychires.2015.06.020

