# Supplementary material for: The effects of high-frequency rTMS over the left DLPFC on cognitive control in young healthy participants
Source: PLoS One. 2017 Jun 14;12(6):e0179430. doi: 10.1371/journal.pone.0179430 (PMC5470713; doi:10.1371/journal.pone.0179430)
Supplement: S1 Table — (PDF) [file pone.0179430.s001.pdf]

S1 Table. The mean N2 amplitudes of the Stroop task under two conditions at two time points in the rTMS and sham rTMS groups.

Group	**mean N2 amplitudes of the Stroop task(uv)**			
	Congruent		Incongruent	
	T1	T2	T1	T2
rTMS group	6.91±3.100	1.87±4.268 [e]	6.70±5.129	1.95±4.162 [e]
Sham rTMS group	6.76±4.732	5.40±4.341	6.67±2.701	5.13±4.931

T1, at baseline, before stimulation; T2, immediately after 7 days of rTMS or sham rTMS.

[e] rTMS vs. sham rTMS: $p<0.05$.
