# Supplementary material for: The effects of high-frequency rTMS over the left DLPFC on cognitive control in young healthy participants
Source: PLoS One. 2017 Jun 14;12(6):e0179430. doi: 10.1371/journal.pone.0179430 (PMC5470713; doi:10.1371/journal.pone.0179430)
Supplement: S2 Table — (PDF) [file pone.0179430.s002.pdf]

S2 Table. The mean N450 amplitudes of the Stroop task under two conditions at two time points in the rTMS and sham rTMS groups.

Group	mean N450 amplitudes of the Stroop task(uv)			
	Congruent		Incongruent	
	T1	T2	T1	T2
rTMS group	9.61±4.804	9.24±5.367	6.59±5.754	2.64±5.798 [f]
Sham rTMS group	9.74±5.614	9.23±5.568	6.96±6.313	6.86±6.980

T1, at baseline, before stimulation; T2, immediately after 7 days of rTMS or sham rTMS.

[f] rTMS vs. sham rTMS: $p<0.05$.
